# Biliatresone induces cholangiopathy in C57BL/6J neonates

**DOI:** 10.1038/s41598-023-37354-z

**Published:** 2023-06-29

**Authors:** Hans Christian Schmidt, Johanna Hagens, Pauline Schuppert, Birgit Appl, Laia Pagerols Raluy, Magdalena Trochimiuk, Clara Philippi, Zhongwen Li, Konrad Reinshagen, Christian Tomuschat

**Affiliations:** Research Laboratory W23, Department of Pediatric Surgery, University Medical Center Hamburg-Eppendorf, Martinistr. 52, 20246 Hamburg, Germany

**Keywords:** Paediatric research, Medical research, Experimental models of disease, Nutrition, Environmental impact

## Abstract

Exposure to plant toxins or microbiota that are able to digest common food ingredients to toxic structures might be responsible for biliary atresia (BA). An isoflavonoid, biliatresone is known to effectively alter the extrahepatic bile duct (EHBD) development in BALB/c mice. Biliatresone causes a reduction of Glutathione (GSH) levels, SOX17 downregulation and is effectively countered with N-Acetyl-L-cysteine treatment in vitro. Therefore, reversing GSH-loss appears to be a promising treatment target for a translational approach. Since BALB/c mice have been described as sensitive in various models, we evaluated the toxic effect of biliatresone in robust C57BL/6J mice and confirmed its toxicity. Comparison between BALB/c and C57BL/6J mice revealed similarity in the toxic model. Affected neonates exhibited clinical symptoms of BA, such as jaundice, ascites, clay-colored stools, yellow urine and impaired weight gain. The gallbladders of jaundiced neonates were hydropic and EHBD were twisted and enlarged. Serum and histological analysis proved cholestasis. No anomalies were seen in the liver and EHBD of control animals. With our study we join a chain of evidence confirming that biliatresone is an effective agent for cross-lineage targeted alteration of the EHBD system.

## Introduction

Biliary atresia (BA) is an inflammatory and fibrotic affection of the extrahepatic (EHBD) and intrahepatic bile ducts (IHBD). The cause of BA puzzles scientists and clinicians since more than two centuries and its molecular progression is not completely understood^1^. There is evidence of an EHBD obstruction^2^. The initial obliteration of the EHBD interrupts bile flow while hepatocytes produce bile constantly. This leads to pre-stenotic dilatation and further damage of the cholangiocytes, which is linked to toxic bile acid accumulation, loss of cell integrity in the EHBD. Periductal bile leakage results in fibrotic response and secondary fulminant obstruction progressing toward the IHBD^3,4^. In the liver toxic bile acid accumulation leads to cytokine release, immune cell recruitment, inflammation, fibrosis and irreversible liver damage^5^. As a result BA is the leading cause of liver transplantation in children and if left untreated affected newborns die within 2 years^6^. Early diagnosis and Kasai hepatoportoenterostomy (KP) lead to restoration of bile flow and reduction of liver damage. The probability for native liver survival was increased if KP was performed before 30 to 45 days postpartum depending on source^7–9^. Screening methods, such as the stool color card and blood analysis for conjugated bilirubin are associated with early diagnosis and lower risk of liver transplantation leading to a live-saving and cost-effective outcome^10,11^.

The incidence of BA varies around the globe, but is higher in the Asia and Pacific region than in Europe and Canada^12^. There are many possible contributing factors, including genetics and prenatal, perinatal and postnatal environmental factors, such as viruses or toxins^13^. Rigid genetic predisposition was excluded and clinical research found no ethnic or familiarity disposition link, but a somewhat higher incidence in females^14,15^. Numerous reports and clinical trials observing the presence of viruses in neonates with confirmed BA produced variable and non-reproducible outcomes^16^.

Currently the opinion tends to environmental factors, such as toxins. A toxin causing isolated EHBD-BA in humans was not confirmed yet. However, an isoflavonoid contained in Australian plants of the dysphania-species, known as biliatresone was observed to affect EHBDs of newborn sheep. Biliatresone selectively induced EHBD-BA in zebrafish and BALB/c mice^17,18^.

Since it is highly conserved among vertebrates, SOX17 (SRY-Box Transcription Factor 17) is essential for controlling endodermal development and maintaining the defined endoderm. The EHBD develops SOX17-dependently and SOX17-deletion results in the loss of biliary structures^19,20^.

It is known that the isoflavonoid biliatresone produces cell membrane instability between cholangiozytes by downregulating the transcription factor SOX17 via a linear signaling cascade involving N-Acetyl-L-cysteine (NAC), Glutathione (GSH), RhoU and Hey2. NAC-treatment counteracts the biliary damage caused by biliatresone and is part of current BA-associated clinical studies^21–23^. Reduced Heat Shock Protein 90 (HSP 90) mediates an extra destabilizing effect. These results support the idea that environmental factors like biliatresone can specifically and potentially cause EHBD-BA^24^.

## Results

### Determination of effective biliatresone amount in C57BL/6J mice

**Figure 1:**
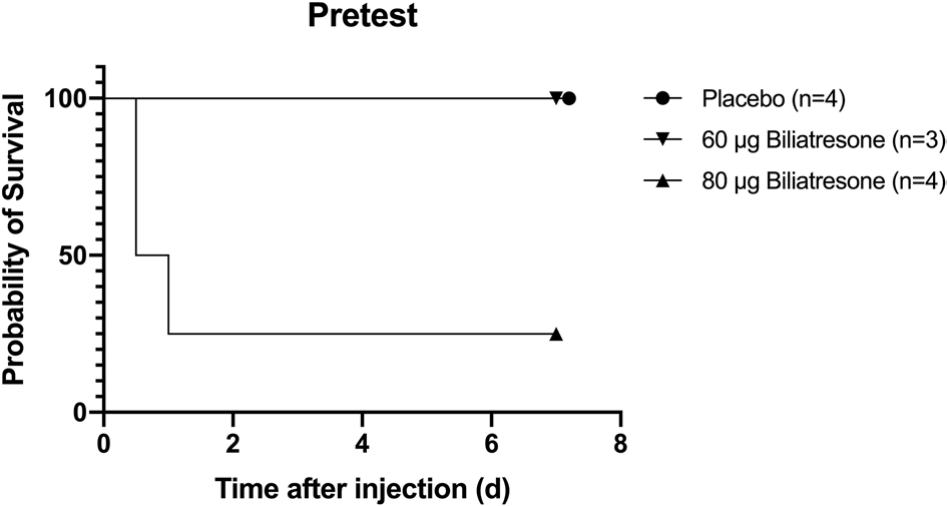
The application of 80 µg biliatresone led to 75% mortality (n = 3) and 100% (n = 3) survival in a group treated with 60 µg biliatresone or DMSO-injection. Surviving individuals developed no signs of cholestasis.

**Figure 2:**
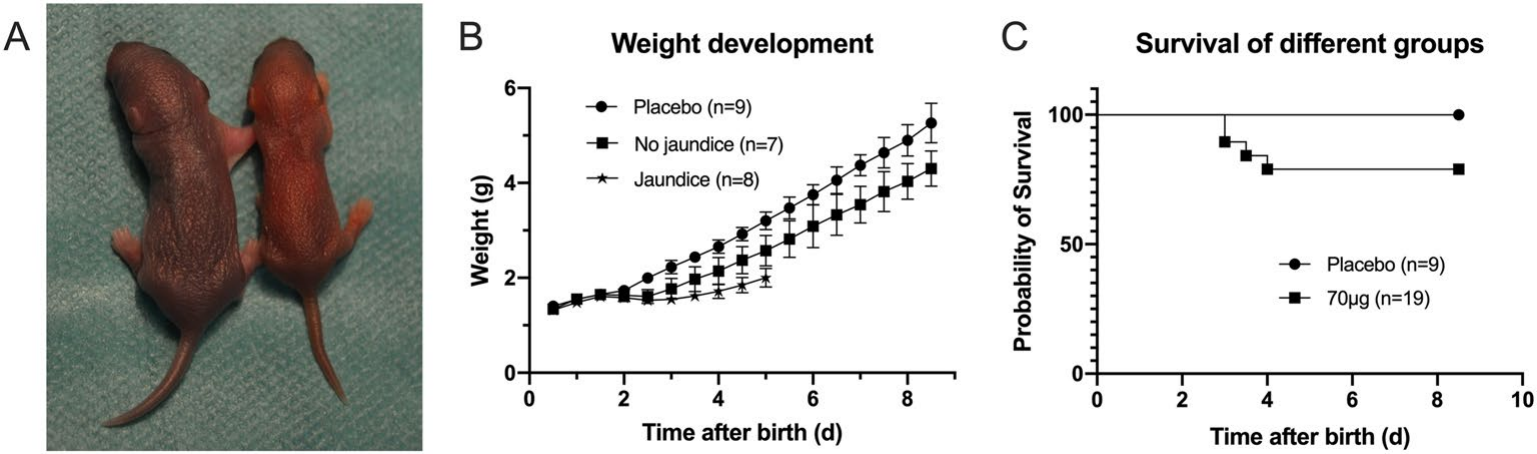
C57BL/6J mice Day 5. Injection of biliatresone induced clinical manifestations of BA in 42.1% (n = 8) including jaundice, acholic stool and delayed weight gain (right) compared to control animal (left) (**A**) Weight development was significantly different between Placebo group and treated mice. Jaundiced mice gained less weight than treated mice with no clinical signs of BA (**B**). After the injection of 70 µg 79% (n = 15) of the individuals survived (**C**).

In a pretest (n = 11) the application of 80 µg was evaluated^18^. In C57BL/6J mice the amount of 80 µg leads to death in 75% (n = 3) of treated neonates. After that investigation a second group (n = 3) was treated with 60 µg biliatresone, and all treated animals survived without clinical signs of cholestasis. All control animals (n = 4) survived indicating no injection- or DMSO related mortality (Fig. 1). 70 µg was determined as the optimal amount of biliatresone and was injected intraperitoneally with a final concentration of 10 µg/µl within the first 36–48 h postpartum. The Survival proportion of the animals treated in the Placebo group in the pretest (n = 4) and in the main experiment (n = 9) was 100% (Figs. 1 and 2C). Therefore mortality in the treatment group could be directly correlated to the toxic effect of biliatresone.

### 70 µg biliatresone induces clinical symptoms in C57BL/6J mice as seen in BA

After determination of time of birth and randomization of the neonates (n = 28) the intraperitoneal injection was performed 36–48 h postpartum. The injection of 70 µg biliatresone in C57BL/6J mice (n = 19) and control solution (n = 9) was performed. Clinical signs of BA only occurred in biliatresone-treated individuals and appeared no earlier than 2 days after the injection. More than 42% (n = 8) of the treated animals developed jaundice (Fig. 2A), ascites, clay-colored stools, yellow urine and delayed weight gain (Fig. 2B) with chance of survival of 79% (n = 15) (Fig. 2C). However 58% (n = 11) of the neonates treated with 70 µg biliatresone either developed without jaundice and discolored stool in 37% (n = 7) or died 21% (n = 4) (Fig. 2B,C).

### Time and weight during injection is crucial for the outcome

**Figure 3:**
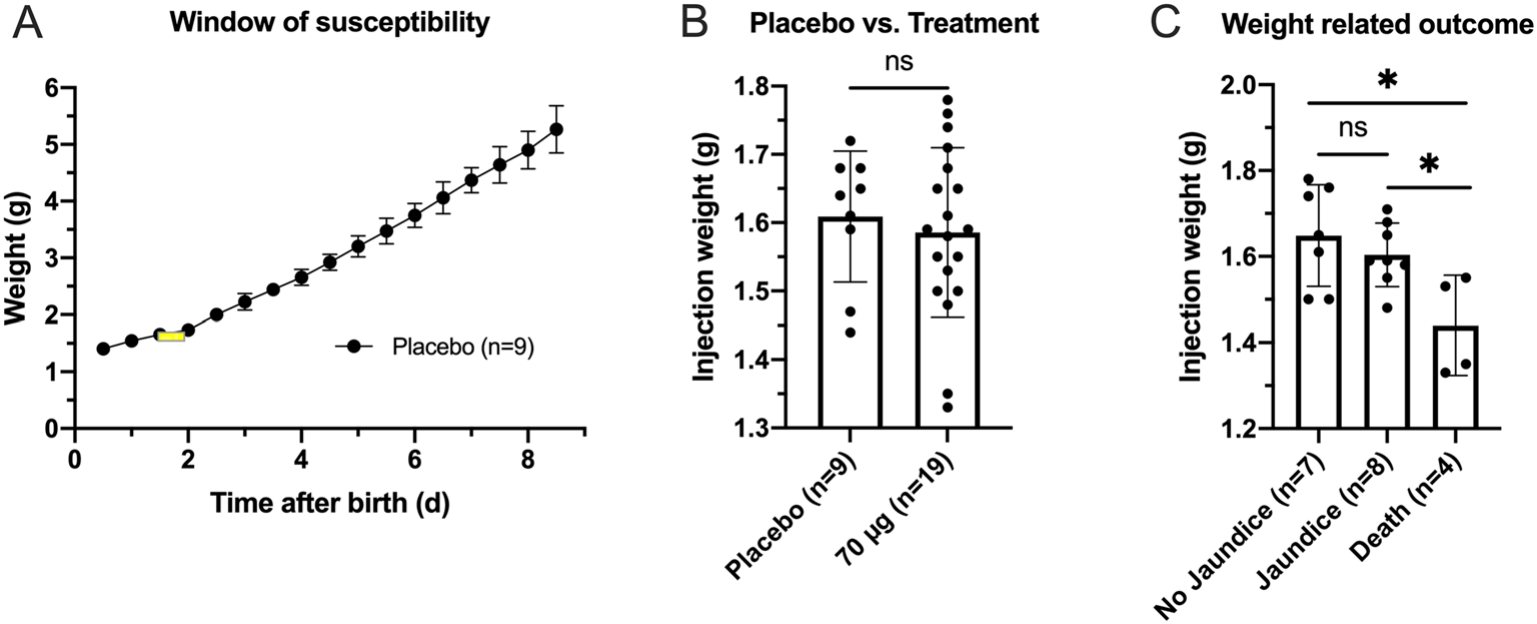
Window of susceptibility (yellow) laid upon the curve of the average weight development of all animals of the Placebo group (**A**) Randomization prior application led to no significant average weight differences (**B**). The outcome after the application of 70 µg biliatresone depends on the injection weight. Animals in the treatment group with the outcome of no signs of cholestasis had an average weight of 1.64 g (n = 7). The weight of animals developing clinical signs of cholestasis had an average weight of 1.60 g (n = 8). An average injection weight of 1.44 g was observed in pups that died within 2 days after the injection (n = 4) (**C**).

The enclosed time of injection in this study and acquired data about the dependence of injection weight and outcome can be interpreted as the optimal window of biliatresone-susceptibility and moment of intraperitoneal injection in C57BL/6J mice (Fig. 3A,C). Control- and treatment group were randomized prior to injection leading to no significant differences at the time of injection (Fig. 3B).

### Serum analysis revealed significant bile and liver specific differences indicating cholestasis

**Figure 4:**
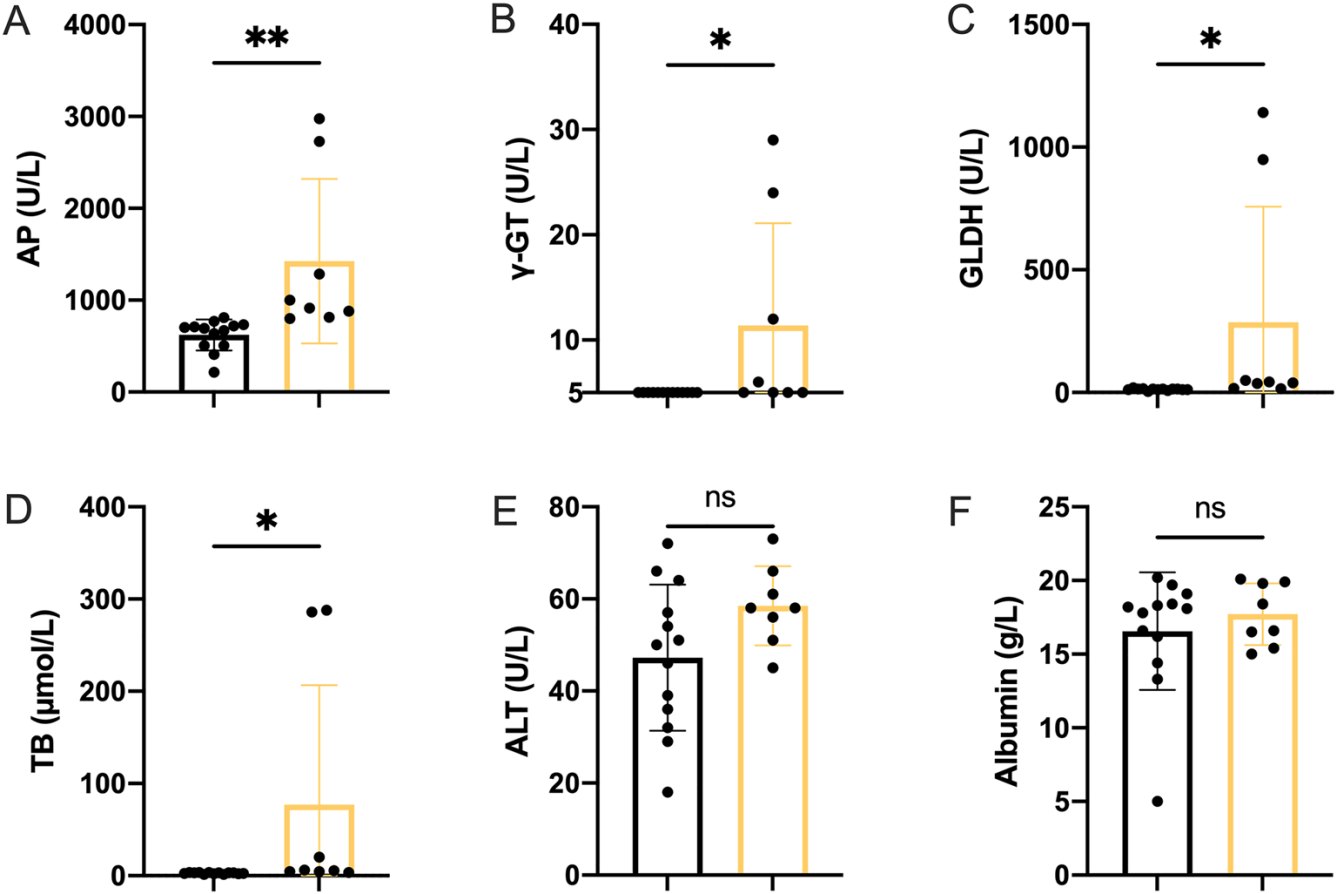
Serum analysis between Placebo group (n = 13, left) and jaundiced individuals (n = 8, right) (**A–F**) alkaline phosphatase (AP) (**A**) gamma-glutamyl transferase (γ- GT) (**B**) glutamate dehydrogenase (GLDH) (**C**) total Bilirubin (TB) (**D**) alanine aminotransferase (ALT) (**E**) Albumin (**F**) *p < 0.05, **p < 0.01. Unpaired t-Test analysis (GraphPad Prism 9).

Analysis of serum via photometry shows significant increases in AP (*p* = 0.0048), γ-GT (*p* = 0.0266), GLDH (*p* = 0.0466), TB (*p* = 0.0483) in biliatresone-treated individuals compared to controls. Contrary, neither ALT (*p* = 0.0820) nor Albumin (*p* = 0.4632) resulted in significant differences (Fig. 4).

### Gross morphology

**Figure 5:**
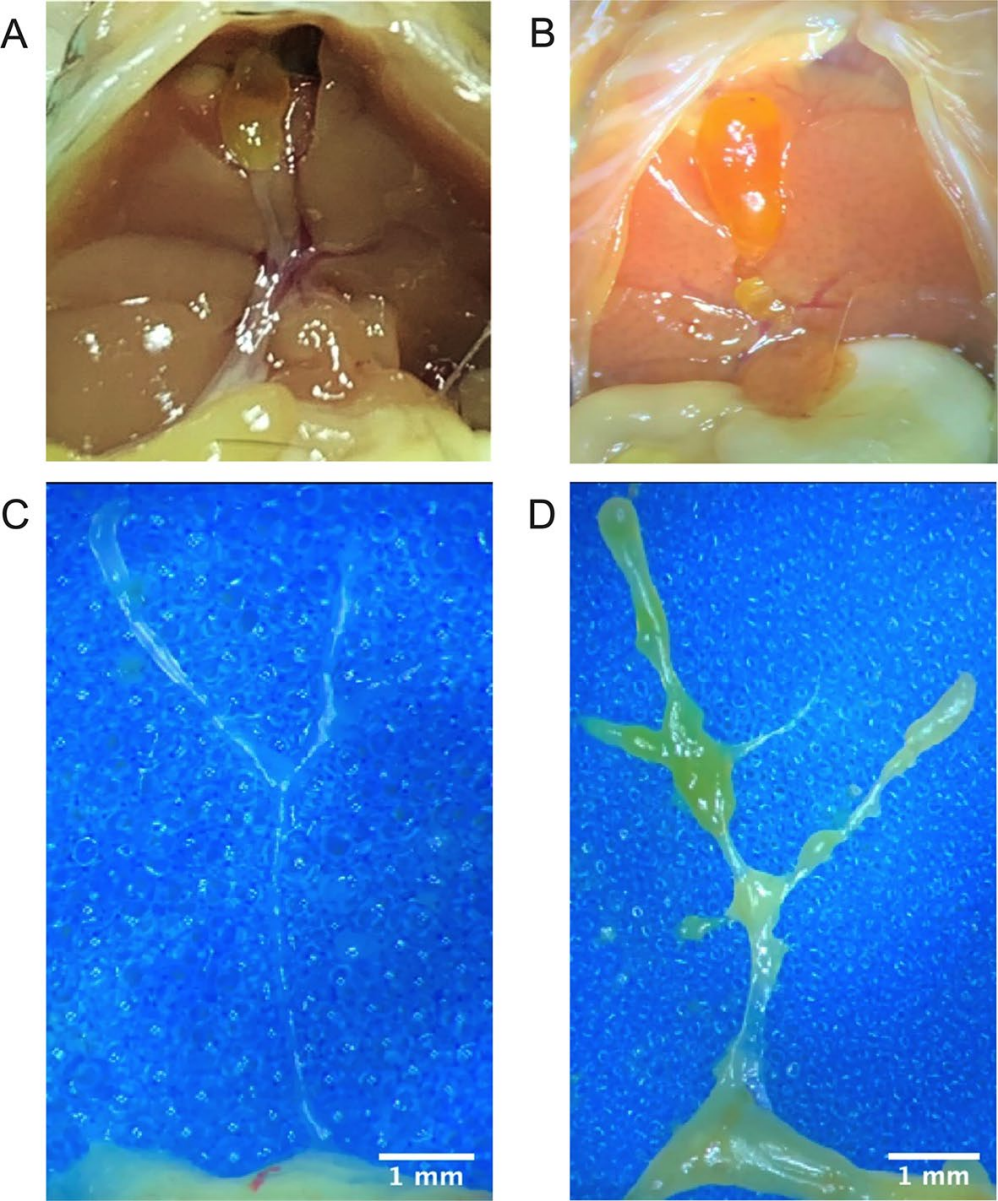
Gross morphology of an intact bile duct (**A**) and twisted and enlarged EHBD and hydropic gall bladder of a jaundiced 5-day old C57BL/6J mice after treatment with 70 µg biliatresone (**B**) Dissection of the EHBD revealed clearly defined borders of animal of the control group (**C**) compared to treated samples with unstructured borders and flanged tissue (**D**).

Intact bile duct system was seen in control animals (Fig. 5A). Surgical dissection of the gallbladders of neonates with persistent jaundice revealed hydropic gall bladders. Additionally, EHBDs were twisted and enlarged (Fig. 5B). Harvested and dissected EHBDs show clean bile duct borders in control samples (Fig. 5A,C) compared to treatment group with flanged tissue (Fig. 5D). The tissue samples in Fig. 5C and 5 D were prepared on a blue foam sponge and examined under a light microscope.

### Histological tissue alterations caused by biliatresone and cholestatic milieu

**Figure 6:**
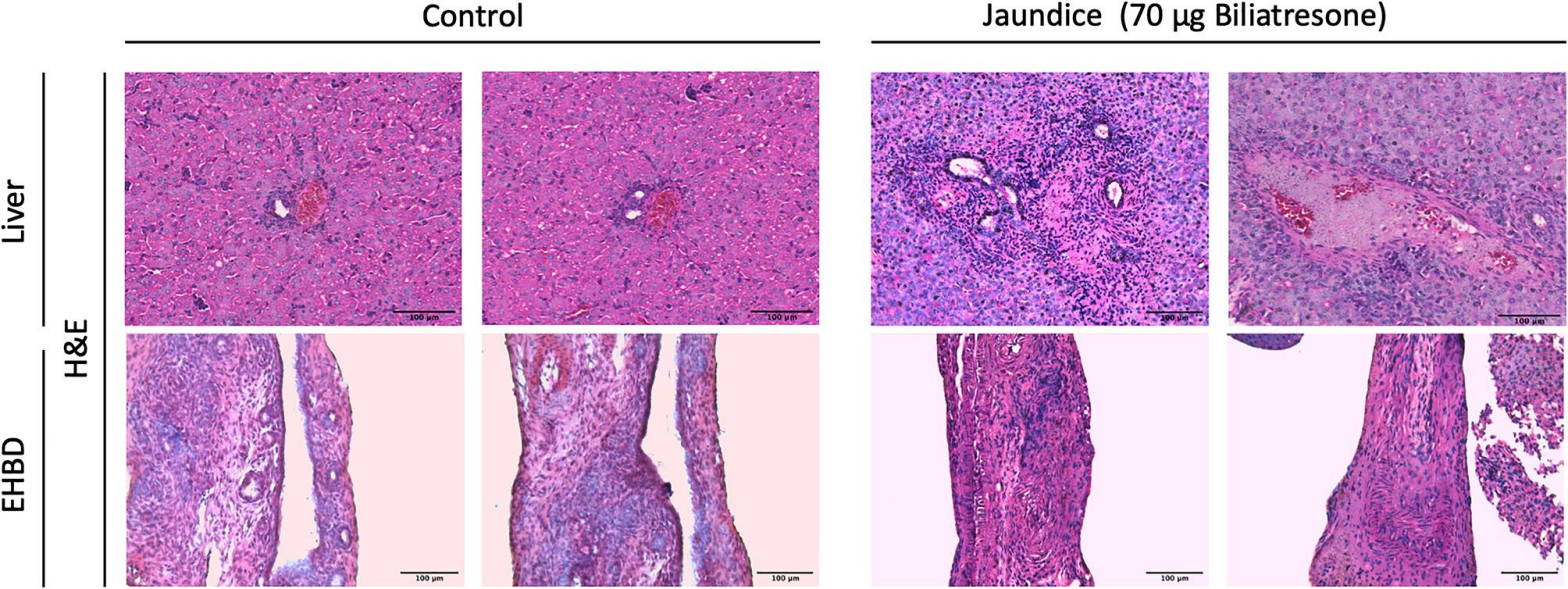
HE staining of the Liver and EHBD: HE staining of the treatment group revealed an absence of lumen in the EHBD and extended periportal fields in the liver. In the control samples an open lumen in the EHBD and no anomalies in the livers could be observed. Scale bars: 100 µm for EHBD and liver.

Liver and EHBD were histologically examined by means of Hematoxylin Eosin (HE) staining. An absence of lumen was observed in EHBDs of jaundiced mice. Microscopy of periportal fields demonstrated extension and infiltration of inflammatory cells (Fig. 6). No developmental anomalies were observed in the livers of the control group and the EHBDs revealed an open lumen.

## Discussion

Although BA is the most common reason for pediatric liver transplantation, little is known about its pathophysiology. Recent years have seen the emergence of new pathomechanisms that have thrown light on environmental toxins as the isoflavonoid biliatresone^25^. In the present study we document for the first time, that biliatresone has an effect on the hepatobiliary tract of C57BL/6J mice. Biliatresone application resulted in clinical symptoms, morphological and histological features comparable to human BA. Jaundice, growth retardation, clay-colored feces and stained urine were all evident in this model (Fig. 2A,B). Additionally, serum studies showed distinct variations in liver- and bile-alteration between the control group and the mice given 70 µg of biliatresone. Cholestatic milieu causes liver damage as seen by significantly higher levels of alkaline phosphatase (AP), gamma-glutamyl transferase (γ-GT) and Glutamate dehydrogenase (GLDH) (Fig. 4A–C)^26,27^. A higher level of total bilirubin (TB) suggests a reduction of liver’s metabolic function (Fig. 4D)^28^. Further evidence for cholestatic pattern is characterized by an increase in AP and TB that is in disproportion to the non-significant difference in Alanine aminotransferase (ALT) (Fig. 4E)^29^. Liver injury is associated with hypoalbuminemia, but serum analysis showed no significant differences in albumin production (Fig. 4F), suggesting no significant liver injury^30^. Similar albumin levels can be explained by early exclusion of animals with clinical signs of cholestasis, thus preventing liver damage. Due to early euthanasia and investigation of jaundiced animals we were able to evaluate very early onset of the biliatresone-mediated effect, which presented similar to a choledochal cyst^2^. Gross morphology of biliatresone treated murine neonates revealed a hydroptic gall bladder and an enlarged, twisted EHBD indicated BA-like phenotype (Fig. 5A,B). By histological examination of EHBD stained with HE, the extrahepatic obliteration was confirmed. The presence of a liver portal extension strengthened the case for cholestasis.

BALB/c mice, the most commonly used strain in hepatobiliary disease modelling was treated with biliatresone in the study of Yang et al. and developed jaundice in 60% including death (20%) ^24^. The overall jaundice rate in biliatresone-treated C57BL/6J mice in the present study was 42.1% (n = 8). Treated animals that did not develop jaundice had a mortality rate of 28.9% in BALB/c mice as opposed to 21.0% (n = 4) in C57BL/6J mice. In the study of Yang et al. ^18^ the total mortality of neonates treated with 80 µg biliatresone was 48.9%, which is compared to a mortality of 11.0% in C57BL/6J mice treated with 70 µg biliatresone much higher, but similar to the mortality in our pretest 75% (n = 3) in neonates treated with 80 µg biliatresone. Regarding the injection we noticed a window of susceptibility consisting of weight, time and dose, which was found to determine the prognosis (Figs. 1 and 3A) concurring with the study of Yang et al.

The postnatal clinical manifestation is consistent with the suspected perinatal onset in human BA. The observed time-dependent efficacy reinforces the reports of neonatal susceptibility in organoids and mice^31^. Since BA only occurs in newborns, it can be assumed that the vulnerability in humans is developmental and exposure to a toxin, such as biliatresone leads to the development of BA. These results are consistent with the murine Rhesus-Rotavirus (RRV) model and the dose- and time dependent morphological disruption of the EHBD in zebrafish after biliatresone exposition^1,32^. The virus kind, virus quantity and injection time point have been reported as being critical in the RRV model for the induction of BA-like phenotype^33,34^.

Since biliatresone has only been evaluated in vivo in BALB/c mouse strains so far, the discovery that we were able to show BA-like characteristics in C57BL/6J strain is quite intriguing. It is well known that BALB/c and C57BL/6J mice differ in their immune characteristics for the defense against infections and outcome. Cross-strain comparison of our data to the study of Yang et al.^18^ leads to the suggestion that the mechanism of action of biliatresone might be independent of the immunological background. C57BL/6 mice are described to be highly resistant compared to BALB/c mice as reported in numerous infectious models^32,35–39^. Different dominance of T-cell response indicate different dealing with antigens^40,41^. C57BL/6 mice display a Th1-dominant immunological response and have been associated with a more robust innate immune response and stronger pro-inflammatory cytokine production (IFN-γ and IL-2). The Th2-dominant immune response of BALB/c mice involves higher levels of IL-4 and IL-10 production and weaker innate immune response, but a stronger adaptive immune response characterized by higher antibody production ^42–44^.

As the evaluation and comparison of the immunological background in the biliatresone-induced BA-like model is not finished yet, future study designs should include treatment under equal conditions. Euthanasia should be performed on determined days in both strains to evaluate BA-like phenotype at the same stages of development. Yang et al. euthanized their animals on day 18 extending the phenotype profile after exhibiting clinical symptoms, which separates the 60% of mice with BA in death (20%), recovery (22.2%) and jaundice for more than 2 weeks (17.8%). This cross-strain comparison shows higher similarity in the biliatresone-induced model than the percentual results in RRV studies. In a comparative study applying the RRV model 5 out of 37 (13.5%) C57BL/6J mice were affected by symptoms of BA after receiving RRV injection. On the other hand, 37 out of 55 BALB/c mice (67%) developed BA-like cholangiopathy and further studies reported up to 86%^32,34,45^. The preferred murine RRV model was not found to accurately simulate BA in all its clinical diversity^46^. It remains questionable whether viral induced BA-like models are suitable for translational research in BA^47^. In addition bench to bed translation of the successful intravenous immunoglobulin (IVIG)-treatment in the RRV-model is questioned by clinical data showing non-significant differences after IVIG treatment^48,49^.

The BA-like cholangiopathy is caused by biliatresone as the controls were treated equally despite biliatresone. Whether biliatresone had a direct impact on the cholangiocytes via the highly conserved SOX17 signal cascade and/or indirectly activated other impact routes leading to inflammation should be investigated in future research. Biliatresone causes GSH-depletion triggering an intracellular linear signaling cascade through upregulation of RhoU and Hey2, finally downregulating SOX17. SOX17 is fundamental for the embryonic development of the EHBD^19^. This formation can be effectively reduced by time-dependent application of biliatresone with a maximum up to 3 days after birth leading to the assumption that a SOX17-developmental window of susceptibility of the EHBD is hit. This cascade is highly conserved and has been documented in zebrafish and BALB/c mice as well as various cholangiocyte cultures and leads to bile duct obstruction^23,50–54^. GSH, a potent antioxidant, is involved in protecting cholangiocytes from oxidative stress. NAC-treatment might be a promising tool to encounter toxic GSH-reduction and additionally mitigate liver damage^22,51^.

Biliatresone is also suggested to downregulate the repair protein HSP90^24^. The measurement of the critical activity of SOX17 and/or HSP90 to prevent and induce BA, is a very exciting idea, should be included in a potential study design for future research and regarded as a promising target for treatment. SOX17- and HSP90-targeting could be performed by use of specific antibodies and analysis in Immunohistochemistry, Immunofluorescence and Western Blot analysis. HSP90 has also been quantified by fluorescent reporter analysis^55^. However, it should be noticed that the regulation of these molecules is complex and likely involves multiple pathways, so further investigation of the molecular mechanisms underlying the effects of biliatresone on BA development would be needed to fully understand their role in BA.

As bile acids are synthesized from cholesterol and facilitate lipid secretion as well as absorption, they also serve as signaling molecules in regulating body metabolism. Conjugation of bile acids to glycine or taurine is mediated by bile acid-CoA amino acid N-acyl- transferase (BAAT) enzymes^56^. Mice and humans differ in the enzymatic repertoire of glycine-conjugation. Human liver bile acids are conjugated with glycine or taurine. Contrary, mice BAATs favor taurine-conjugation and are particularly inefficient at conjugating glycine^57^. In rodents, the substrate Chenodeoxycholic acid (CDCA) of the glycine-conjugated bile acid glycochenodeoxycholic acid (GCDCA) is metabolized dominantly to muricholic Acids (MCAs)^58^. During cholestasis, as shown in the bile duct ligation model, a paradox increase of the production of bile acids, especially MCA but not glycine-conjugated bile acids is reported^59^. Interestingly, if the murine bile acid pool is humanized with hydrophobic GCDCA an increased occurrence of cholestasis and cirrhosis has been observed^60^. Upregulation of glycine-conjugated bile acids is associated with increased cholangiocyte damage and their periductal leakage is linked to periductal fibrosis as a result of periductal fibroblast overactivation followed by overshooting collagen deposition and luminal obstruction^3,61^. Therefore it can be assumed that humans are more prone to develop cholangiopathies. Of interest, in BA patients´ dried blood samples GCDCAs were significantly elevated compared to jaundiced controls and GCDCA is the most accumulated bile salt in human cholestasis^62,63^. Consequently, the question arises whether biliatresone does not also affect the enzymes of the murine bile acid mechanism.

Recently, Gupta et al. (2023) applied biliatresone (15 µg/g) orally in pregnant BALB/c-mothers on the days 14 and 15 post mating. Elevated levels of glycine-conjugated bile acids, including GCDCA in combination with a lack of clinical symptoms of BA were reported. In that regard it seems likely that the incidence of mild hepatobiliary injury may be common and much higher than that of severe injury and associated with the very low incidence of BA^64^.

It is reasonable to expect that rodents and humans will always be exposed to low dosage hazardous precursors of toxins. If the effect is not increased by the immature organism's susceptibility, different doses of such precursors may not necessarily alter development of healthy newborns and cholangiocyte integrity and they do not go on to acquire obstructive cholangiopathies such as BA. Neonatal exposure to environmental toxins may happen intrauterine or during nursing. However, it is currently unknown, which internal or external factor is most likely to cause postnatally induced EHBD-BA in newborn infants^65^. Retracing digestive potential of the microbiota it was observed that Rhizoctonia solani inoculated sugar beet roots take on a structure resembling biliatresone and the bacterium Clostridium sporogenes can break down a soybean isoflavone (Daidzein) into biliatresone^66–68^. Plants use phytoalexins, such as the isoflavonoids for the defense against microbiota. These are linked to several disease-promoting and -affecting progressions in humans and might be consumed during pregnancy probably having toxic effects after ingestion^69^. Additionally low maternal intake of copper, beta tocopherol, vitamin E and phosphorus was associated with the occurrence of isolated BA^70^.

Whether rodent models are suitable for translational research to monitor therapeutic effects of glycine-conjugated bile acids in murine neonates is questionable as their enzymatic repertoire differs decisively from the human one. Therefore 3D-cellculture based on human primary tissue might be a promising alternative to best evaluate involved described and alternative pathways. As bile acid deficiency causes dysbiosis^71^ future studies should also focus on the interaction between microbial capacity and bile acid metabolisms.

## Methods

### Animal experimental model

The study was carried out on a cohort of newborn C57BL/6J mice (n = 39), including biliatresone-treated (n = 26) individuals. In order to validate biliatresone-mediated pathology effects DMSO-injected controls were assessed as negative controls (n = 13). Both male and female neonates were used for all analyses. C57BL/6J adult animals were obtained from Jackson Laboratory (USA). Neonates were kept together with their one-to-one mated parent animals in individually ventilated cages (IVC). The environmental conditions were controlled in temperature (20–24 °C), 12:12 h light–dark cycle and relative humidity of 40–70%.

**Figure 7:**
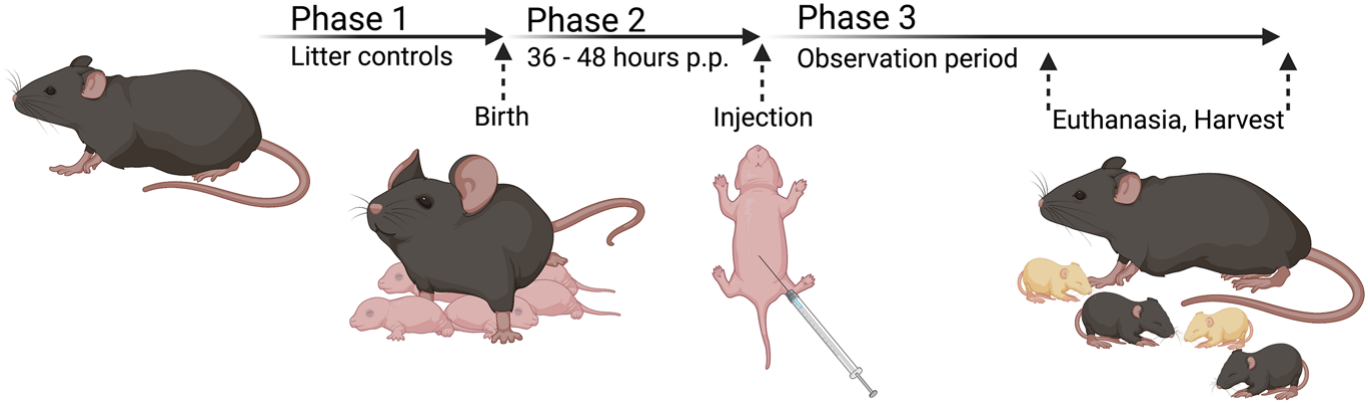
Model of biliatresone application in murine C57BL/6J mice. Phase 1 describes litter controls to determine the time of birth. Phase 2 includes 36–48 h postpartum (p.p.) ending with the injection of biliatresone. Phase 3 depicts an observation period up to day nine or the onset of clinical signs of cholestasis followed by euthanasia and harvest. Created with Biorender.com.

This study aimed for a biliatresone-mediated BA in C57BL/6 mice following on from a previous study using BALB/c mice^18^. As shown in Fig. 7 pregnant C57BL/6J mice were observed at least twice a day to determine the time of birth (Phase 1). After randomization the intraperitoneal injection was performed 36–48 h postpartum (Phase 2). Neonates were scored until day nine or the onset of clinical signs of cholestasis, such as jaundice, delayed weight gain, clay-colored stools and discolored urine. The experiment was completed with decapitation and the removal of the liver and gall bladder (Phase 3).

### Reagents and toxins

Biliatresone was solved in DMSO as recommended by the manufacturer (CS- 0,068,115, ChemScene, New Jersey, USA) to a concentration of 100 mg/ml and stock aliquots were stored at − 80 °C. This stock solution was diluted in saline, containing 0.5% Tween-20 (37,470.01, Serva, Heidelberg, Germany) to a working solution with a final concentration of 8.57 µg/µl for the application of 60 µg, 10 µg/µl for the application of 70 µg or 11.43 µg/µl for the application of 80 µg prior application. 7 µl of that working solution were injected intraperitoneally in C57BL/6J mice within the first 36–48 h postpartum. A saline-based solution containing the same amount DMSO as in the treatment solution served as a control. Proportion between treatment and control group was 2:1.

### Techniques

The intraperitoneal injection was performed using a 0.5 ml syringe with a 30 G canula of 8 mm length (324,825, BD Medical, Allschwil, Switzerland). The needle was slowly inserted in the umbilicus nearly parallel to the skin and directed to the upper right abdomen followed by slow injection and withdrawal. EHBD and liver were harvested using the En-bloc-Resection for murine neonatal EHBDs^72^. In brief, the access to the abdomen was ensured. Cutting of pyloric region of the stomach and duodenum right lateral of the duodenal papilla was performed. Remaining structures connecting the liver to the abdomen were dissected and the En-Bloc-sample was transferred to a foam mat where the liver and EHBD samples were isolated. Tissue samples were immediately fixated in Paraformaldehyde (4% PFA in PBS) after harvesting. The samples were stored for 6 h (EHBD) or 24 h (Liver) at four degrees, embedded in paraffin, stored at room temperature and cut in 2 µm-slides prior to staining. Slides were deparaffinized in Rotihistol and rehydrated in a descending alcohol row, stained each 10 min for Hematoxylin (41-5130-00, Medite) and Eosin (41-6660-00, Medite), dehydrated in an ascending alcohol series and covered afterwards. The stained samples were observed using broad field microscopy.

### Serum analysis

Decapitation was followed by trunk blood collection. Collected blood was centrifuged (1500 rpm, 10 min, 21 °C) and the serum was collected and stored at − 80 °C. Serum analysis of alkaline phosphatase (AP), gamma-glutamyl transferase (γ-GT), Glutamate dehydrogenase (GLDH), Total Bilirubin (TB), Alanine aminotransferase (ALT) and Albumin was performed (synlab.vet, Geesthacht, Germany) by means of photometry.

### Statistical analyses

Statistical analyses were generated using GraphPad Prism 9 (San Diego, CA, USA). Results were presented as the mean ± standard deviation using the student’s t test. *P* values < 0.05 were considered statistically significant.

### Ethical approval and institutional review board statement

Procedures involving animal subjects have been approved by the Institutional Animal Care and Use Committee (IACUC) at the University Medical Center Hamburg-Eppendorf (N045/21). All methods were performed according to the institutional animal care, relevant guidelines and regulations. The study is reported in accordance with the ARRIVE guidelines.

## Supplementary Information

Supplementary Information.

## Data Availability

Raw data were generated at (University Medical Center Hamburg-Eppendorf, Hamburg, Germany, Department of Pediatric Surgery). Derived data supporting the findings of this study are available from the corresponding author (C.T., H.C.S.) on request.

## Abbreviations

BA: Biliary atresia
EHBD: Extrahepatic bile duct
IHBD: Intrahepatic bile ducts
GSH: Glutathione
NAC: *N*-Acetyl-l-cysteine
SOX17: SRY-box transcription factor 17
HSP 90: Heat shock protein 90
AP: Alkaline phosphatase
γ-GT: Gamma-glutamyltransferase
GLDH: Glutamate dehydrogenase
TB: Total bilirubin
ALT: Alanine aminotransferase
RRV: Rhesus-Rotavirus
HE: Hematoxylin eosin staining
IVIG: Intravenous immunoglobulin
MCA: Muricholic acids
GCDCA: Glycochenodeoxycholic acid
