# Supplementary figures and images for: Biliatresone induces cholangiopathy in C57BL/6J neonates
Source: Sci Rep. 2023 Jun 29;13:10574. doi: 10.1038/s41598-023-37354-z (PMC10310722; doi:10.1038/s41598-023-37354-z)

Supplementary Figure 1  
Overview pedigree of the animal numbers used

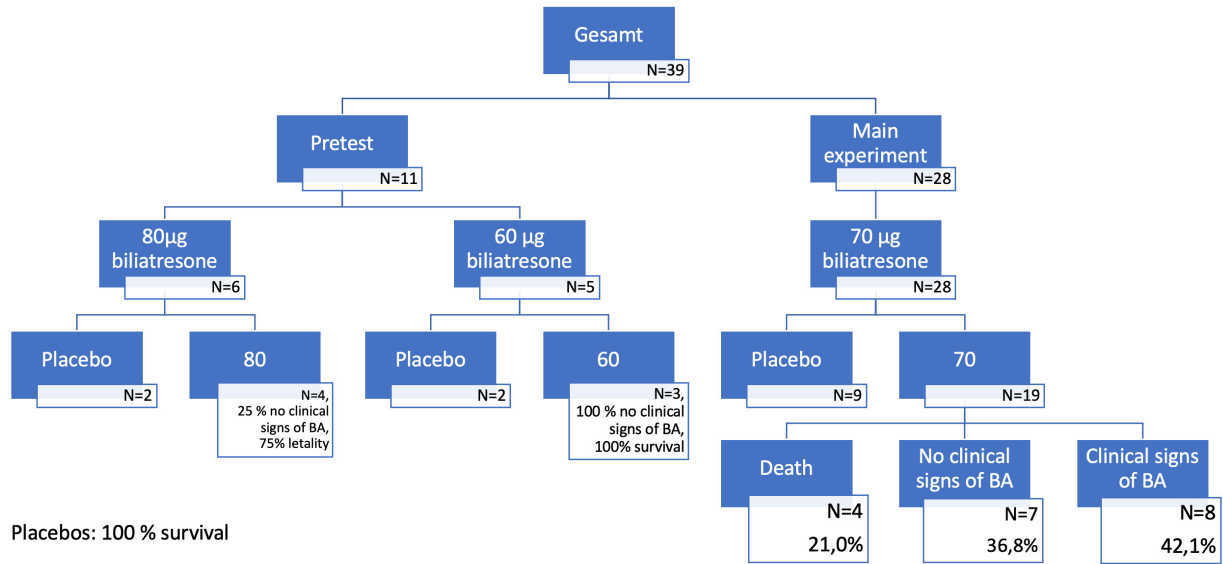

Supplement: Supplementary file 1 — Supplementary Information. [file 41598_2023_37354_MOESM1_ESM.pdf]
